# A quantitative risk assessment to evaluate the efficacy of mitigation strategies to reduce highly pathogenic avian influenza virus, subtype H5N1 (HPAI H5N1) in the Menoufia governorate, Egypt

**DOI:** 10.1186/s12917-021-02917-7

**Published:** 2021-06-07

**Authors:** Yumna Elsobky, David Nganwa, Gamal El Afandi, Ahmed Byomi, Gopal Reddy, Ehsan Abdalla

**Affiliations:** 1 Department of Hygiene and Zoonosis, Faculty of Vet. Medicine, University of Sadat City, Sadat City, Menofia, 32897 Egypt; 2 Department of Pathobiology/Department of Graduate Public Health, College of Veterinary Medicine, Tuskegee University, Tuskegee, AL 36088 USA; 3 College of Agriculture, Environment and Nutrition Sciences, Tuskegee University, Tuskegee, AL 36088 USA; 4 Department of Astronomy and Meteorology, Faculty of Science, Al-Azhar University, Cairo, 11884 Egypt; 5 Department of Pathobiology, College of Veterinary Medicine, Tuskegee University, Tuskegee, AL 36088 USA; 6 Department of Graduate Public Health, College of Veterinary Medicine, Tuskegee University, Tuskegee, AL 36088 USA

**Keywords:** Egypt, HPAI-H5N1, Menoufia governorate, Monte Carlo simulations, Prevalence, Quantitative risk assessment, Sensitivity analysis

## Abstract

**Background:**

The poultry industry in Egypt has been suffering from endemic highly pathogenic avian influenza (HPAI) virus, subtype H5N1 since 2006. However, the emergence of H9N2, H5N8, and H5N2 in 2011, 2016, and 2019 respectively, has aggravated the situation. Our objective was to evaluate how effective are the mitigation strategies by a Quantitative Risk Assessment (QRA) model which used daily outbreak data of HPAI-H5N1 subtype in Egypt, stratified by different successive epidemic waves from 2006 to 2016.

**Results:**

By applying the epidemiologic problem-oriented approach methodology, a conceptual scenario tree was drawn based on the knowledgebase. Monte Carlo simulations of QRA parameters based on outbreak data were performed using @Risk software based on a scenario-driven decision tree. In poultry farms, the expected probability of HPAI H5N1 prevalence is 48% due to failure of mitigation strategies in 90% of the time during Monte Carlo simulations. Failure of efficacy of these mitigations will raise prevalence to 70% with missed vaccination, while failure in detection by surveillance activities will raise it to 99%. In backyard poultry farms, the likelihood of still having a high HPAI-H5N1 prevalence in different poultry types due to failure of passive and active surveillance varies between domestic, mixed and reservoir. In mixed poultry, the probability of HPAI-H5N1 not detected by surveillance was the highest with a mean and a SD of 16.8 × 10–3 and 3.26 × 10–01 respectively. The sensitivity analysis ranking for the likelihood of HPAI-H5N1 in poultry farms due to missed vaccination, failure to be detected by passive and active surveillance was examined. Among poultry farms, increasing vaccination by 1 SD will decrease the prevalence by 14%, while active and passive surveillance decreases prevalence by 12, and 6%, respectively. In backyard, the active surveillance had high impact in decreasing the prevalence by 16% in domestic chicken. Whereas the passive surveillance had less impact in decreasing prevalence by 14% in mixed poultry and 3% in domestic chicken.

**Conclusion:**

It could be concluded that the applied strategies were not effective in controlling the spread of the HPAI-H5N1 virus. Public health officials should take into consideration the evaluation of their control strategies in their response.

**Supplementary Information:**

The online version contains supplementary material available at 10.1186/s12917-021-02917-7.

## Introduction

The poultry industry in Egypt has been experiencing endemic highly pathogenic avian influenza (HPAI) virus, subtype H5N1 (HPAI-H5N1) since 2006 [1]. The situation has been aggravated by the emergence of H9N2, H5N8, and H5N2 in 2011, 2016, and 2019 respectively [2–4]. With continuous circulation and long term endemicity for more than a decade, H5N1 viruses have predictably undergone substantial genetic evolution [5], which resulted in increased binding ability of the virus to the human receptor [6, 7]. This resulted in Egypt reporting the highest number of human cases per country worldwide [5, 8, 9]. Along with the changed HPAI-H5N1 virus pathogenicity pattern in poultry, mortality increased up to 100% in poultry flocks in the first wave of 2006 then dropped to 20–60% afterwards [5, 10].

Egyptian authorities have made constant efforts to mitigate the distress, since the early control strategies after the HPAI-H5N1 virus introduction, which included: increasing awareness; stamping out infected birds (within 3 km of the initial outbreak); surveillance; banning live bird markets; quarantine by restriction of movement within a 7 km radius from the outbreak location and emergency vaccination of parent flocks [1, 10]. Not all of these, however, limited the spread of infection. Therefore, the decision was made to increase vaccination to cover all commercial flocks and backyard poultry [1]. From then onwards, control strategy changed to mainly mass vaccination, surveillance, and preemptive culling of infected birds to combat the disease [11]. Despite these control efforts applied by the government, HPAI-H5N1 became endemic by 2008 with continuous and extensive circulation revealed by the regular nationwide active, passive, and targeted surveillance activities [11–17].

Vaccination has become the main tool used to control the HPAI-H5N1 virus in Egypt, as other aspects of the control strategies became neglected, including biosecurity [18]. Using vaccination became a routine, and Egypt is considered as the second country after China which accounted for over 99% of vaccination usage for highly pathogenic avian influenza [19]. It is noticeable that mass vaccination is not effective if not complemented by a correct outbreak management, bio-security measures, and does not reach an adequate coverage [20–22]. As a consequence, the efficiency of vaccination was reduced over time with vaccine failures that have occurred following the emergence of antigenic drift variants [23]. Therefore, inadequate vaccination policies could drive antigenic drift rather than control [5].

Egypt has become an epicenter for A(H5) virus evolution, and outbreaks in poultry continued to occur with a genetic drift in the hemagglutinin (HA) gene observed each year [5, 9]. What constituted a challenge to effectively control virus spread and infection, besides poultry industry infrastructure itself, is a great obstacle for control of the disease [23]. Before deciding on the continued use of vaccination for controlling HPAI, the outcome must be compared with that of other control measures, in order to make a better use of existing resources [5, 18]. It is an urgent requirement for decision-makers to reach define a more concerted, controlled, and regulated strategy than what has been done to date. To the authors’ knowledge, this is the first study to evaluate the mitigation strategies in Egypt using a Quantitative Risk Assessment (QRA) model which used daily outbreak data of HPAI-H5N1stratified by different Epidemic waves from 2006 to 2016. This work presents a pilot study in Menoufia governorate, Egypt.

## Materials and methods

### The epidemiologic problem-oriented approach (EPOA)

EPOA is a methodology that facilitates the development of systematic and structured knowledge bases which provides the essential framework for model development. The methodology is very useful and essential in the collection and analysis of epidemiological information and data and comprises two basic steps: (i)problem identification and (ii) problem solving. Using the EPOA, a knowledgebase was developed after reviewing several relevant precedent peer-reviewed studies, coupled with data collected from different sources [24–26].

The QRA methods are extensions of standard statistical and epidemiological methods [27], which are expressed numerically and enable one to evaluate the likelihood and consequences of an adverse event occurring [28]. In a QRA, each parameter requires to be described and scientific evidence presented for the justification of the parameter estimates. In the present study, a QRA was developed to examine the likelihood of poultry to became infected with the HPAI-H5N1 virus due to failure of mitigation strategies such as vaccination, passive and active surveillance. This had been applied and stratified by six epidemic waves ***(EW1-EW6)*** from 2006 to 2016, in the Menoufia Governorate, Egypt. The quantitative and qualitative definition of “epidemic wave in Menoufia Governorate” was illustrated by Elsobky and colleagues [29]. This QRA model relied on outbreaks daily data collected by national authorities stratified by six epidemic waves that occurred in this period. The quantified parameter input values presented in terms of probability distributions were: total number of poultry affected; non-infected poultry; and prevalence rates.

We considered all HPAI H5N1 outbreaks that occurred in the six epidemic waves in Menoufia governorate since the introduction of the virus from 2006 to 2016. The assumptions we made are mentioned below, in addition to those provided in supplementary files. In each epidemic wave EW (EW1- EW6)), prevalence rates (PR) of infection in whole poultry populations were calculated. The total poultry population of each epidemic wave (EW) was calculated relative to the period in which the EW lasted. Since the total population in each EW was not the same, prevalence was standarized by multiplying them by 100,000 poultry population for comparison of the different PRs of the EWs. Under the policy of mass vaccination as a mitigation strategy in poultry farm (we assume that all the population is vaccinated), we expect that vaccine protection help in infection reduction, except for missed poultry farms, which are still susceptible and at risk of getting infection with influenza A subtype HPAI H5N1 virus (not successfully protected by vaccine; not properly vaccinated; not vaccinated at all). When both type of surveillance (active and passive) are applied in both domestic poultry and backyard poultry farm, we assumed that surveillance will successfully detect infected poultry population and remove them. This would help in infection reduction except for those not detected by any kind of surveillance, which will allow the virus circulation and the emergence of new epidemic waves, with high prevalence rates despite the application of mitigation strategies.

**Fig. 1:**
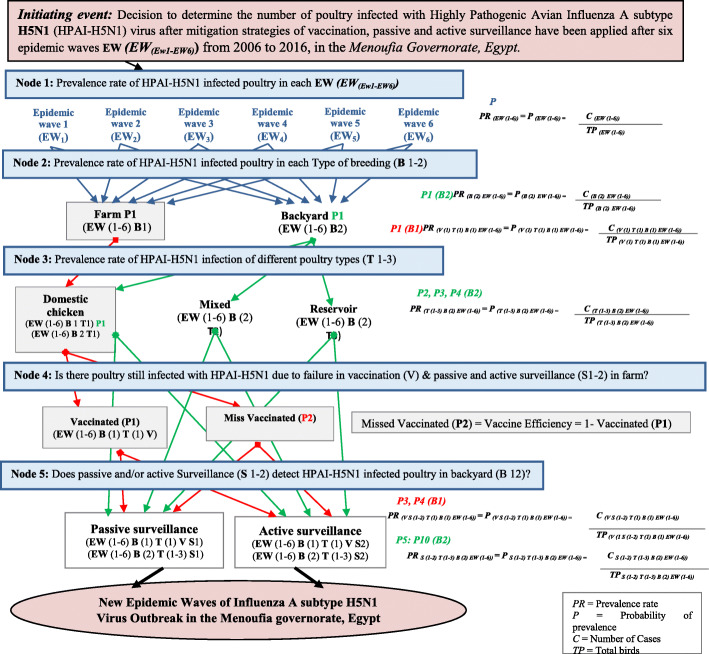
Risk pathway (Scenario tree) for the likelihood of HPAI-H5N1 prevalence rate in poultry due to missed vaccination, not detected by surveillance in six epidemic waves

**Fig. 2:**
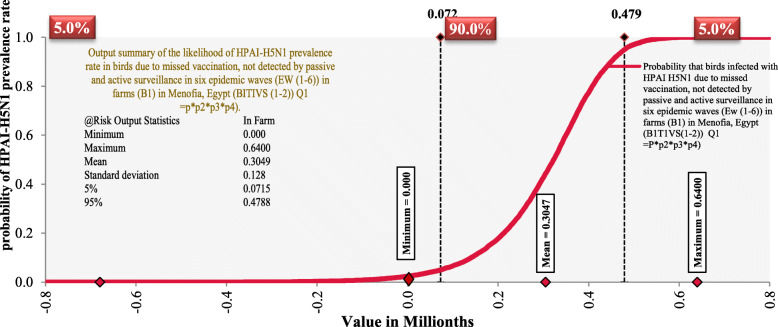
A sigmoid curve showing the probability of HPAI-H5N1 prevalence rate in poultry due to missed vaccination, not detected by surveillance in six epidemic waves in farms (B1)

The risk pathway (Scenario Tree) presented in Fig. 1, consists of a sequence of specific events and for each node or event a specific question related to the risk of transmission of HPAI-H5N1 is asked. Values for each parameter using the collected data for each node are tabulated. Using the appropriate @Risk function and simulation, a probability distribution is determined for that specific parameter. The product of these probability distributions of the answers to these questions will determine the final risk related to the likelihood of transmission of HPAI-H5N1 virus due to missed vaccination or failure to detect sick poultry by passive or active surveillance. The variables are organized into five major categories used as parameter estimations (Fig. 2). Monte Carlo simulations with iterations set at 10,000 iterations for the QRA input parameters of HPAI-H5N1 infection transmission due to missed vaccination, failure to detect sickly poultry by passive or active surveillance was implemented with @Risk software version 5.7 (Palisade Corporation). We considered the multiplier effect of raw data following 10,000 Monte Carlo iterations, which had some limitations on the number of points that had to plotted in a chart. Sensitivity analysis was used to show the aggregate effect of each input variable on the likelihood of missed vaccination and failure to detect sick poultry by passive or active surveillance activities on HPAI-H5N1 infection transmission. At each of the nodes, probability distributions were assigned and @ Risk Bestfit distributions together with Monte Carlo simulation were used in determining the efficacy of vaccination and failure to detect sick poultry by passive or active surveillance in reducing HPAI-H5N1 infection transmission in the poultry farms and the backyard poultry. A tornado graph was used to depict the sensitivity of HPAI H5N1 transmission (output) to changes in mitigation strategies (inputs) using regression and correlation coefficients.

In the sensitivity analysis, regression and correlation coefficients were both used to indicate the direction of the relationship whether positive or negative. A positive relationship exists when the output increases as the input parameter increases; and a negative one when the output decreases while input increases. A regression coefficient shows the strength of the relationship, and a correlation coefficient shows the consistency of the relationship. The coefficient of determination *(R*^*2*^*)* is used to decide whether to choose correlation coefficients or regression coefficients. A low value of *R*^*2*^ means that a linear regression model is not very good at predicting the output from the indicated inputs. In this case, the focus is more on correlation coefficients, because rank-order correlation does not depend on the two distributions having a similar shape or being linearly related. If *R*^*2*^ is high, a linear regression model is a good mathematical fit [30].

### Assumptions

The following assumptions were made when developing the QRA about the decision of determining the number of infected poultry with HPAI-H5N1 virus after mitigation strategies of vaccination and surveillance had been applied for six epidemic waves (EW (Ew1-EW6)) from 2006 to 2016, in the Menoufia Province, Egypt.

1. In this study, the model is built on the domestic poultry farms and the backyard poultry considering the two main divisions of the poultry industry in Egypt [11]. Most of the production takes place in sector 3 (small commercial farms) and sector 4 (village or backyard poultry) in Egypt according to the Food and Agriculture Organization (FAO) classification [5, 19].
2. All poultry population in a given single village in Menoufia governorate, Egypt, was considered infected with HPAI-H5N1 virus when one outbreak was detected within a certain circumscribed location in this village at a certain point in time, according to Toma and colleagues [31], who defined an outbreak as ‘the confirmed presence of disease, clinically expressed or not, in at least one bird in a defined location and during a specified period of time’.
3. A village is the smallest epidemiologic unit in Menoufia governorate, and each village contains 62,316 poultry on average based on the total poultry population, from which the total number of cases is calculated for each outbreak event.
4. The total poultry population of each epidemic wave (EW) was calculated relative to the period in which the EW lasted, and the prevalence rate was then calculated per 100,000 poultry population.
5. Any poultry species raised in the backyard other than domestic chickens are considered reservoir poultry, while domestic chickens raised with any other species are considered mixed poultry.
6. Domestic chickens is the most common type raised in the commercial sector in Egypt [23]. All outbreaks data of farms were obtained after vaccination by the poultry producers, considering the highly variable standards applied (different vaccines, frequency, dose, route, age, etc.) and the lack of monitoring of the vaccination programme by the Egyptian veterinary services [1, 12].
7. In Egypt, vaccine failures have occurred following antigenic drift in field viruses [22]. The immune pressure exerted by the vaccines or natural infection accelerates virus evolution in poultry, reducing the efficacy of vaccination over time [23, 32–37]. Besides, also improper administration, mishandling [38] and inappropriate storage of the vaccine [12], or suppression of the immune system (i.e.: due to chicken anemia virus infection or ingestion of mycotoxins) [39] can lead to reduced efficacy of the vaccine. All these factors could be considered as missed vaccinated poultry.
8. There is no available data indicating vaccination of backyard poultry,. This highlight that vaccination coverage of household poultry is lower than 20%. Therefore, vaccination of the backyard poultry is no longer provided nor supervised by the government. A previous study showed that village cumulative annual flock immunity (CAFI) from household vaccination carried out by the Egyptian government is unlikely to be maintained at the levels required to significantly reduce the virus load and restrict transmission [18].
9. Notification and surveillance of poultry infected with the HPAI-H5N1 virus reported by owners were considered as passive surveillance. While targeted surveillance or preslaughter or live bird market (LBM) surveillance samples were considered as active surveillance. All surveillance data were considered under active and passive surveillance terms as defined in previous studies [13, 15].

### Evidence gathering

The evidence underlying this QRA comes from published data, studies, routine reports, and other technical documents from public health organizations and agencies including the Food and Agriculture Organization of the United Nations (FAO), World Health Organization (WHO), and Centers for Disease Control and Prevention (CDC). This evidence was collected and documented for each node of the scenario tree. Appropriate probability distributions were assigned to the various nodes. These probability distributions capture the variability and uncertainties associated with each event occurring on the scenario tree. Appropriate probability distributions were also used to represent the prevalence rates of HPAI-H5N1 outbreak.

### Study area

Egypt is in the northeast corner of Africa, spanning approximately 1 million square kilometers. As per United Nations estimates, the human population of Egypt is 100 million, with most of them living in the Nile Delta [5], where there were recorded higher disease incidences as a reflection of high densities of poultry and human activities [40, 41]. This pilot study was carried out using the extent of one of the Nile Delta governorates (Menoufia, Egypt), where the highest number of outbreaks were recorded [5, 40]. In addition to that, Menoufia is considered the leading poultry producing governorate in Egypt [42], district level is the smallest administrative unit used for defining surveillance and control strategies related to HPAI (H5N1) among poultry [43].

### Data source

Domestic chicken HPAI-H5N1 outbreak data used in this study were extracted from the Egyptian ministry of agriculture (Egyptian Committee for Veterinary Services) official reports for national surveillance for the study period from January 2006 to December 2016.

### Parameter estimations

#### Initiating event

IE Decision to determine the number of poultry infected with Highly Pathogenic Avian Influenza A subtype H5N1 (HPAIH5N1) virus after mitigation strategies of vaccination, passive and active surveillance has been applied after six epidemic waves ***EW (EW (EW1-EW6))*** from 2006 to 2016, in the Menoufia, governorate, Egypt.

### N - The total number of poultry population in Menoufia, governorate Egypt in each epidemic wave

#### Description

The data that was collected is historical and not well organized to facilitate capture of the variability and uncertainty of the total number of poultry in six epidemic waves ***EW (EW (Ew1-EW6))*** whereby the available data were given in intervals. In a QRA using Monte Carlo simulation, uncertain inputs in a model are captured by using ranges of possible values known as probability distributions. Using probability distributions, input variables can be expressed as probability distributions for the different outcomes occurring. Probability distributions are a much more realistic way of describing uncertainty in variables of a risk analysis [44].

#### Probability distribution

The probability distributions of individual input variables at each node were determined by using the @Risk Bestfit distributions (See Tables 1 and 2 for details). @Risk Bestfit distribution was used to pick the best distribution of the data whereby the variability and uncertainty of prevalence rate of HPAI-H5N1virus infection would be best captured.

**Table 1:** Parameter estimates for a quantitative risk assessment of HPAI-H5N1 in poultry farms (B1) in Menoufia, Egypt. For all the parameter estimates, we used the best-fit probability distributions

Parameter notation	Parameter description	Best-fit probability distributions	Minimum	Maximum	Mean	Std Dev	5%	95%
**N**	Total number of poultry in Menoufia, Egypt	***RiskPareto*** (0.72898,3,749,375.0)	3,749,375	52,857,042	23,511,705	22,775,444	3,749,374	52,857,042
**P**	The probability of HPAI-H5N1 prevalence rates in poultry in each epidemic wave	***RiskExtValueMin*** (0.44313,0.11949)	0.08253	0.55095	0.36933	0.17576	0.08253	0.55095
**P1**	The probability of HPAI-H5N1 prevalence rates in poultry that were vaccinated in farms	***RiskLaplace*** (0.10269,0.053750)	0.01651	0.1791	0.0988	0.05487	0.01651	0.1791
**P2** **(1-P1)**	The probability of HPAI H5N1 prevalence rates in birds that missed vaccination in farm							
**P3a**	The probability of HPAI-H5N1 prevalence rates in poultry due to detection by passive surveillance in farms	***RiskExpon***(0.024769, ***RiskShift***(−0.0041281))	0	0.066481	0.024769	0.026972	0	0.066481
**P3** **(1-P3a)**	The probability of HPAI H5N1 prevalence rates in birds due to failure in detection by passive surveillance in farm							
**P4a**	The probability of HPAI-H5N1 prevalence rates in poultry due to detection by active surveillance in farms	***RiskUniform*** (−0.0077277,0.15365)	0.01533	0.1306	0.07403	0.04811	0.01533	0.1306
**P4** **(1-P4a)**	The probability of HPAI H5N1 prevalence rates in birds due to failure in detection by active surveillance in farm

**Table 2:** Parameter estimates for a quantitative risk assessment of (HPAI-H5N1) in backyard poultry (B2). For all the parameter estimates, we used the best-fit probability distributions

Parameter notation	Parameter description	Best-fit probability distributions						
		Function	Minimum	Maximum	Mean	Std Dev	5%	95%
**N**	*Total number of poultry in Menoufia, Egypt*	***RiskPareto*** (0.72898,3,749,375.0)	3,749,375	52,857,042	23,511,705	22,775,444	3,749,374	52,857,042
**P**	*The probability of HPAI-H5N1 prevalence rates in poultry in each epidemic wave*	***RiskExtValueMin*** (0.44313,0.11949)	0.08253	0.55095	0.36933	0.17576	0.08253	0.55095
**P1**	*The probability of HPAI-H5N1 Prevalence rates in Backyard poultry (B2) in each EW*	***RiskUniform*** (−0.0058329,0.49715)	0.06602	0.42529	0.26682	0.1612	0.06602	0.42529
**P2**	*The probability of HPAI-H5N1 Prevalence rates in domestic chicken in backyard*	***RiskUniform*** (−0.023407,0.25598)	0.01651	0.21606	0.10608	0.08142	0.01651	0.21606
**P3**	*The probability of HPAI-H5N1 Prevalence rates in mixed poultry in backyard*	***RiskUniform*** (−0.032087,0.21864)	0.00373	0.18282	0.09288	0.08264	0.00373	0.18282
**P4**	*The probability of HPAI-H5N1 Prevalence rates in reservoir poultry in backyard*	***RiskExpon*** (0.067856, Risk Shift (−0.011309))	0	0.21171	0.06786	0.08028	0	0.21171
**P5a**	*The probability of H5N1 Prevalence rates in domestic chicken due to detection by passive surveillance in backyard*	***RiskUniform*** (−0.010885,0.090071)	0.003537	0.075648	0.036997	0.031555	0.003537	0.075648
**P5** **(1-P5a)**	*The probability of H5N1 Prevalence rates in domestic chicken due to failure in detection by passive surveillance in backyard*							
**P6a**	*The probability of H5N1 Prevalence rates in domestic chicken due to detection by active surveillance in backyard*	***RiskPareto*** (0.76486,0.012968)	0.01297	0.1662	0.06909	0.0619	0.01297	0.1662
**P6** **(1-P6a)**	*The probability of H5N1 Prevalence rates in domestic chicken due to failure in detection by active surveillance in backyard*							
**P7a**	*The probability of H5N1 Prevalence rates in mixed poultry due to detection by passive surveillance in backyard*	***RiskPareto*** (0.51481,0.0023579)	0.002358	0.099722	0.03821	0.040182	0.002358	0.099722
**P7** **(1-P7a)**	*`The probability of H5N1 Prevalence rates in mixed poultry due to failure in detection by passive surveillance in backyard*							
**P8a**	*The probability of H5N1 Prevalence rates in mixed poultry due to detection by active surveillance in backyard*	***RiskUniform*** (−0.023198,0.13919)	0	0.11599	0.05467	0.04632	0	0.11599
**P8** **(1-P8a)**	*The probability of H5N1 Prevalence rates in mixed poultry due to failure in detection by active surveillance in backyard*							
**P9a**	*The probability of H5N1 Prevalence rates in reservoir poultry due to detection by passive surveillance in backyard*	***RiskExpon*** (0.0080041, RiskShift (− 0.0013340))	0	0.034822	0.008004	0.013668	0	0.034822
**P9** **(1-P9a)**	*The probability of H5N1 Prevalence rates in reservoir poultry due to failure in detection by passive surveillance in backyard*							
**P10a**	*The probability of H5N1 Prevalence rates in reservoir poultry due to detection by active surveillance in backyard*	***RiskExpon*** (0.059852, RiskShift (−0.0099753))	0	0.21171	0.05985	0.07899	0	0.21171
**P10** **(1-P10a)**	*The probability of H5N1 Prevalence rates in reservoir poultry due to failure in detection by active surveillance in backyard*							

### Node 1- P1: period prevalence of HPAI-H5N1 infection in poultry in each EW (EW (EW1- EW6))

#### Period prevalence

As described previously in parameter ***N***, the data in nodes ***1–5*** is also historical and not well organized, to capture the variability and uncertainty of ***PR*** of influenza A subtype HPAI-H5N1 virus outbreak in birds in each epidemic wave ***EW (EW (EW1- EW6))*** where the available data were given in intervals. @Risk Bestfit distributions were used to generate probability distributions. This specific variable is the prevalence rate ***(PR)*** of HPAI-H5N1 infection in poultry expressed as the probability distribution in each epidemic wave ***EW (EW (EW1- EW6))*** stratified by ***P, C, and TP*** respectively **(**See Tables 1 and 2 for details). Thus, both the numerator and denominator in ***PR*** were estimated using Monte Carlo simulations with @Risk software. The probability distribution values for this variable were determined by @RiskBestfit as described previously in parameter ***N***. The general mathematical formula used for calculating the ***PR*** of HPAI-H5N1 infection in poultry in each epidemic wave ***EW (EW (EW1- EW6))*** were calculated using available data on disease cases from 2006 to 2016 in the Menoufia province, Egypt is as follows:

1 $$ {\boldsymbol{P}\boldsymbol{R}}_{\left( EW\ \left(1-6\right)\right)}={\boldsymbol{P}}_{\left( EW\ \left(1-6\right)\right)}=\frac{{\boldsymbol{C}}_{\left( EW\ \left(1-6\right)\right)}}{{\boldsymbol{TP}}_{\left( EW\ \left(1-6\right)\right)}} $$

Whereby ***P*** is the probability distribution, ***C*** is outbreak cases of HPAI-H5N1 infection and ***TP*** is total poultry population, respectively.

### Node 2-P2: prevalence rate (PR) of HPAI-H5N1 infected poultry in each type of breeding (B 1–2) in each EW (EW (EW1-EW6))

This specific variable represents the prevalence rate ***(PR)*** of HPAI-H5N1 infection in the backyard poultry in each epidemic wave ***EW (EW (EW1- EW6)),*** stratified by type of breeding ***(B 1–2)*** and ***P, C, TP*** respectively. The variable input values for this parameter ***P2*** are calculated by dividing the number of cases of HPAI-H5N1 by the total populations in different breeding categories. The probability distribution values for this variable were determined by @RiskBestfit as described previously in parameter ***N*** (See Tables 1 and 2 for details**)**. The general mathematical formula is as follows:

2 $$ {\boldsymbol{P}\boldsymbol{R}}_{\left(B\ \left(1-2\right)\  EW\ \left(1-6\right)\right)}={\boldsymbol{P}}_{\Big(B\ \left(1-2\right)\ \left( EW\ \left(1-6\right)\right)}=\frac{{\boldsymbol{C}}_{\left(B\ \left(1-2\right)\  EW\ \left(1-6\right)\right)}}{{\boldsymbol{TP}}_{\left(B\ \left(1-2\right)\  EW\ \left(1-6\right)\right)}} $$

Whereby ***B1*** is the poultry farms and ***B2*** is the backyard poultry, respectively.

### Node 3-P3: prevalence rate (PR) of HPAI-H5N1 infected poultry in each type of breeding (B 1–2) different poultry types (T 1–3) in each EW (EW (EW1-EW6))

This specific variable represents the prevalence rates ***(PR)*** of HPAI-H5N1 infection in poultry in each epidemic wave ***(EW)*** stratified by type of breeding ***(B 1–2)*** and different poultry types ***(T 1–3)*** and ***P, C, TP***. The variable input values for this parameter ***P3*** are calculated by dividing the number of cases of HPAI-H5N1 by the total populations in different breeding categories and different poultry types to be expressed as probability distributions. The probability distribution values for this variable were determined by @RiskBestfit as described previously in parameter ***N*** (See Tables 1 and 2 for details). The general mathematical formula is as follows:

3 $$ {\boldsymbol{P}\boldsymbol{R}}_{\left(T\ \left(1-3\right)\ B\ \left(1-2\right)\  EW\ \left(1-6\right)\right)}={\boldsymbol{P}}_{\Big(T\ \left(1-3\right)\ B\ \left(1-2\right)\ \left( EW\ \left(1-6\right)\right)}=\frac{{\boldsymbol{C}}_{\left(T\ \left(1-3\right)\ B\ \left(1-2\right)\  EW\ \left(1-6\right)\right)}}{{\boldsymbol{TP}}_{\left(T\ \left(1-3\right)\ B\ \left(1-2\right)\  EW\ \left(1-6\right)\right)}} $$

Whereby ***T1*** is domestic chicken, ***T2 is*** mixed poultry and ***T2*** reservoir poultry, respectively.

### Node 4-P4: are there poultry still infected with HPAI-H5N1 due to failure in vaccination (V) and passive and active surveillance (S 1–2) in the farms in domestic chicken (T 1) in each EW (EW (EW1-EW6))

This specific variable represents the prevalence rate ***(PR)*** of HPAI-H5N1 infection in vaccinated ***(V)*** poultry after passive and active surveillance ***(S 1–2)*** used in each epidemic wave ***EW (EW (EW1-EW6))*** stratified by type of breeding ***(B 1)*** and ***P, C, TP***. The variable input values for this parameter ***P4*** are calculated by dividing the number of cases of HPAI-H5N1 by the total populations in the poultry farms, vaccination and surveillance types. The probability distribution values for this variable were determined by @RiskBestfit as described previously in parameter ***N*** (See Table 1 for details). In this study, it was assumed that only domestic chickens raised in farms were vaccinated. The general mathematical formula is as follows:

4 $$ {\boldsymbol{P}\boldsymbol{R}}_{\left(V\ (1)S\ \left(1-2\right)\ B\ (1)\  EW\ \left(1-6\right)\right)}={\boldsymbol{P}}_{\Big(V\ (1)\ S\left(1-2\right)\ B\ (1)\ \left( EW\ \left(1-6\right)\right)}=\frac{{\boldsymbol{C}}_{\left(V\ (1)\ S\ \left(1-2\right)\ B\ (1)\  EW\ \left(1-6\right)\right)}}{{\boldsymbol{TP}}_{\left(V\ (1)\ S\ \left(1-2\right)\ B\ (1)\  EW\ \left(1-6\right)\right)}} $$

Whereby ***V*** is vaccinated poultry, ***S1*** is passive surveillance and ***S2*** is active surveillance, respectively.

### Node 5-P5: does passive and active surveillance (S 1–2) detect HPAI-H5N1 infected poultry in the backyard (B 2) in different poultry types (T 1–3) in each EW (EW (EW1-EW6))?

This specific variable represents the prevalence rate ***(PR)*** of HPAI-H5N1 infection after passive and active surveillance ***(S 1–2)*** used in each epidemic wave ***EW (EW (EW1-EW6))*** stratified by type of breeding ***(B 2)*** and different poultry types and different poultry types: domestic, mixed and reservoir ***(T 1–3)*** and ***P, C, TP*** respectively. The variable input values for this parameter ***P5*** are calculated by dividing the number of cases of HPAI-H5N1 by the total populations in the backyard poultry, surveillance types, and different poultry types. The probability distribution values for this variable were determined by @RiskBestfit as described previously in parameter ***N*** **(**See Tables 1 and 2 for details**)**. Whereas, in this node ***(Node 5)*** it was assumed that in the backyard poultry no vaccination measures were taken except in a few cases. Therefore, in this study, it was assumed that vaccination has not been used in backyard poultry. The general mathematical formula is as follows:

5 $$ {\boldsymbol{P}\boldsymbol{R}}_{\left(S\ \left(1-2\right)\ T\ \left(1-3\right)\ B\ (2)\  EW\ \left(1-6\right)\right)}={\boldsymbol{P}}_{\left(s\ \left(1-2\right)\ T\ \left(1-3\right)\ B\ (2)\  EW\ \left(1-6\right)\right)}=\frac{{\boldsymbol{C}}_{\left(S\ \left(1-2\right)\ T\ \left(1-3\right)\ B\ (2)\  EW\ \left(1-6\right)\right)}}{{\boldsymbol{TP}}_{\left(S\ \left(1-2\right)\ T\ \left(1-3\right)\ B\ (2)\  EW\ \left(1-6\right)\right)}} $$

Whereby ***S1*** is passive surveillance and ***S2*** is active surveillance, respectively.

We justify ***P1*** as poultry that is 100% properly vaccinated and when they get infection then they can be easily detected and there is no risk in this category, while ***P2*** is considered as poultry that has missed vaccination and are still at risk of getting infection due to many reasons after strategy of mass vaccination (vaccination failure, human error, or vaccine itself or not being vaccinated at all). The infected poultry are not detected and escape mitigation strategies in place for the different poultry breeding types and were assumed to allow the virus to circulate in the environment to interact with poultry and new EW will continue to occur.

#### P3a (B1), P6a, P8a, P10a (B2)

how effective the passive surveillance is, in detecting infected poultry and remove them from population.

#### P3 (B1), P6, P8, P10 (B2)

passive surveillance cannot detect 100% of the infection, Still there is infection that cannot be detected by the passive surveillance. This failure is mainly due to less experience in recognizing symptoms or due to underreporting.

#### P4a(B1), P5a, P7a, P9a (B2)

how effective the active surveillance is, in detecting infected poultry and remove them from population. ***P4 (B1), P5, P7, P9 (B2):*** active surveillance cannot detect 100% of infection, still there is infection that cannot be detected by active surveillance. This failure is due to problems in coverage or in availability of adequate funding.

The input parameters in this study are independent events of each other, the occurrence of one of the events gives us no information about whether or not the other event will occur; that is, the events have no influence on each other. Therefore, the value of the product ***(P x P2 x P3 x P4)*** is for the likelihood of poultry still having a high period prevalence of HPAI-H5N1 (P) due to the failure of the independent mitigation strategies.

## Results

### Poultry farms

As represented by the sigmoid curve in Fig. 2, the results show that in the poultry farms, the likelihood of poultry still having a high prevalence rate of HPAI-H5N1 ***(P)*** after the failure of mitigation strategies such as vaccination ***(P2)***, passive surveillance ***(P3)***, and active surveillance ***(P4)*** in six epidemic waves ***(EW (1–6))*** ranged from 0.0001 to 6.40 × 10^− 01^ with a mean and a standard deviation (SD) of 3.05 × 10^− 03^ and 1.28 × 10^− 01^ respectively. The value of the product ***(P x P2 x P3 x P4)*** is for the likelihood of poultry still having a high prevalence rate of HPAI-H5N1 (P) due to the failure of these mitigation strategies.

In 90% of the time during Monte Carlo simulations, the expected probability of HPAI H5N1 prevalence rate in birds in farms is 48% due to failure of mitigation. It is expected that HPAI-H5N1 prevalence rates in farm will increase to 70% with missed vaccination ***P2*** (*population still at risk after application of mass vaccination*), while in case of failure in detection by surveillance (passive and active) (***P3 and P4***), it will increase up to 99%.

### Sensitivity analysis

**Fig. 3:**
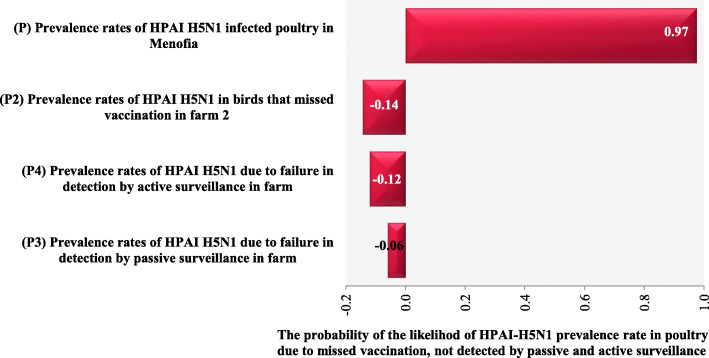
Tornado graph showing the likelihood of HPAI-H5N1 prevalence rate in poultry due to missed vaccination, not detected by surveillance in six epidemic waves in farms (B1)

The sensitivity analysis ranking of regression coefficients of tornado graph (Fig. 3) shows that the likelihood of HPAI-H5N1 prevalence rate has on six epidemic waves ***(EW (1–6))*** in the poultry farms ***(B1)*** in Menoufia, Egypt due to missed vaccination, failure to detect of sickly poultry by passive or active surveillance was examined. In the poultry farms, increasing vaccination will decrease the likelihood of increasing prevalence rates by 14%, in active surveillance by 12%, and in passive surveillance by 6% (regression coefficients = − 0.14, − 0.12 and − 0.06 respectively) **(**Table 3 and Fig. 3**)**. Therefore, vaccination, active, and passive surveillance were considered to be strong predictors of the likelihood of HPAI-H5N1 prevalence due to missed vaccination, and failure to detect sick poultry by passive or active surveillance in poultry farms ***(B1)***.

**Table 3:** Sensitivity ranking for the likelihood of HPAI-H5N1 prevalence rate in poultry due to missed vaccination, not detected by surveillance in six epidemic waves in the poultry farms (B1)

Sensitivity Ranking	Variables	Regression coefficient values
1	*HPAI-H5N1 prevalence rates in poultry in each epidemic wave*	**0.97**
2	*HPAI-H5N1 Prevalence rates in poultry that missed vaccination in farm*	**−0.14**
3	*HPAI-H5N1 prevalence rates in poultry due to failure in detection by active surveillance in farm*	**−0.12**
4	*HPAI-H5N1 prevalence rates in poultry due to failure in detection by passive surveillance in farm*	**−0.06**

As shown in Fig. 3, concerning regression coefficients, the longer the bar or the larger the coefficient, the greater the impact that a particular input has on the corresponding output being analyzed. A positive coefficient, with a bar extending to the right, indicates that this input has a positive impact: increasing this input will increase the output [30]. For example, in this study, it is the case of HPAI-H5N1 prevalence rates in infected poultry in Menoufia in each epidemic wave, with the highest regression coefficient of 0.97. A negative coefficient, with a bar extending to the left, indicates that this input has a negative impact on the output: increasing this input will decrease the output [30]. For example, in this study, HPAI-H5N1 prevalence rates due to failure in detection by passive surveillance in farm poultry with the lowest regression coefficient of − 0.06. The tornado graph **(**Fig. 3**)** shows pictorial representations of sensitivity analyses of the simulation results of the probabilities of HPAI-H5N1 prevalence rates in poultry due to missed vaccination and failure to detect sick poultry by passive or active surveillance. This was in six ***(EW (1–6))*** in the poultry farms in Menoufia, Egypt.

Tornado graph shows how the outputs are ranked by the magnitude that input effects have had on the outputs. In addition, Table 3 shows simulation ranking results in tabular form. The tornado graph shows the influence of how much each specific input distribution has on the change in the values of the corresponding specific output distribution [45].

In addition, Fig. 3 shows the likelihood of HPAI-H5N1 prevalence rates ***(P)*** were higher in poultry in each epidemic wave ***(EW (1–6))***. Whereas HPAI-H5N1 prevalence rates likelihood in poultry were lower due to missed vaccination, failure to detect sickly poultry by passive or active surveillance in the farm ***(P2, P4, and P3)***, as the inputs were less likely affected the output. For instance, in this study, when the prevalence rate parameter ***(p)*** input is changed by one SD given that the other input parameters are held constant, the output will change by 97%. This is the influence that the input will have on the output. In the case of the mitigation inputs, by one SD while holding the other inputs the same the outputs will decrease by the indicated percentages [45]. In this case, increasing vaccination as a mitigation parameter by one SD will decrease the prevalence rates by 14%, while increasing active surveillance by one standard deviation SD, will decrease prevalence rates by 12%. These were followed by passive surveillance, which will affect in decreasing the prevalence rates by only 6% (regression coefficients = − 0.14, − 0.12, and − 0.06 respectively).

### Backyard poultry

**Fig. 4:**
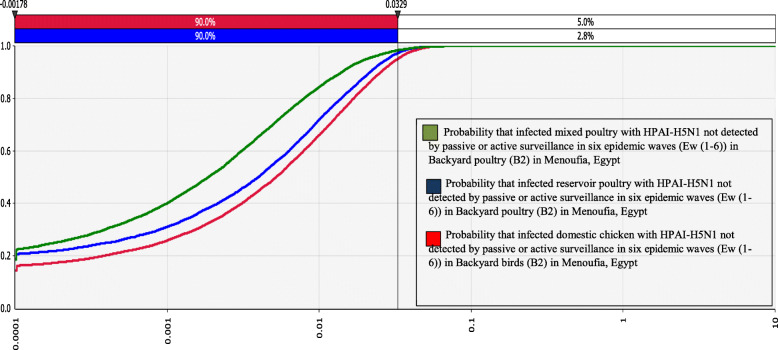
Comparison of cumulative probability distributions of HPAI-H5N1 prevalence rate in different poultry types not detected by surveillance in six epidemic waves in Backyard birds (B2)

Table 4 and Fig. 4, illustrate the results of comparison of cumulative probability distributions (as represented by sigmoid curves) of HPAI-H5N1 prevalence rates in different poultry types not detected by passive and/or active surveillance in six epidemic waves ***(EW (1–6))*** in the backyard poultry ***(B2)***. In domestic poultry, the HPAI-H5N1 prevalence not detected by passive and/or active surveillance ***(P5)*** and ***(P6)*** respectively; in mixed poultry, the HPAI-H5N1 prevalence not detected by passive and/or active surveillance ***(P7)*** and ***(P8)*** respectively; in reservoir poultry, the HPAI-H5N1 prevalence not detected by passive and/or active surveillance ***(P9)*** and ***(P10)*** respectively). The x-axis shows the three overlying graphs which represent the cumulative probability distributions of HPAI-H5N1 prevalence rates while the y-axis shows the probability confidences of the risk value equal to or less than the values on the x-axis [25]. In the backyard poultry, the likelihood of still having a high HPAI-H5N1 prevalence rate in three different poultry types (domestic, mixed, and reservoir) due to failure of passive and active surveillance varies between these three types. In mixed poultry, the value of the product ***(P x P7x P8)*** is the likelihood of poultry still having a high prevalence of HPAI-H5N1 (P) due to the failure of these mitigation strategies. This value was higher, ranging from 0.0001 to 1.06 × 10^− 3^ with a mean and a SD of 16.8 × 10^− 3^ and 3.26 × 10^− 01^ respectively. This was followed by domestic chicken, the value of the product ***(P x P5 x P6)*** is for the likelihood of poultry still having a high prevalence of HPAI-H5N1 (P) due to the failure of these mitigation strategies. This value ranging from 0.0001 to 7.08 × 10^− 01^ with a mean and a SD of 1.48 × 10^− 3^ and 3.69 × 10^− 01^ respectively and in reservoir poultry, the value of the product ***(P x P9 x P10)*** is for the likelihood of poultry still having a high prevalence of HPAI-H5N1 (P) due to the failure of these mitigation strategies. This value ranging from 0.0001 to 1.11 × 10^− 01^ with a mean and a SD of 4.94 × 10^− 3^ and 8.34 × 10^− 03^ respectively. However, the likelihood of HPAI-H5N1 prevalence in mixed poultry in the backyard not detected by passive or active surveillance is 3.4 times per 100,000 poultry population higher than those of reservoir poultry and 113 times per 100,000 poultry population higher than those of domestic chicken.

**Table 4:** Output summary statistics for the likelihood of HPAI-H5N1 prevalence rate in different poultry types not detected by surveillance in six epidemic waves in Backyard poultry (B2)

@RISK Output statistics	Probability of HPAI-H5N1 prevalence rate in domestic chicken not detected by passive or active surveillance in six epidemic waves (EW (1–6)) in Backyard poultry (B2)	Probability of HPAI-H5N1 prevalence rate in mixed poultry not detected by passive or active surveillance in six epidemic waves (EW (1–6)) in Backyard poultry (B2)	Probability of HPAI-H5N1 prevalence rate in reservoir poultry not detected by passive or active surveillance in six epidemic waves (EW (1–6)) in Backyard birds poultry (B2)
Minimum	0.0001	0.0001	0.0001
Maximum	7.08 × 10^−01^	1.06 × 10^− 03^	1.11 × 10^− 01^
Mean	1.48 × 10^−3^	16.8 × 10^−3^	4.94 × 10^−3^
SD	3.69 × 10^− 01^	3.26 × 10^− 01^	8.34 × 10^− 03^
5%	0.0001	0.0001	0.0001
95%	3.29 × 10^−3^	2.83 × 10^− 3^	2.08 × 10^− 3^

### Sensitivity analysis

Likewise, in the poultry farms, the sensitivity analysis ranking of the tornado graph for the likelihood of decrease of HPAI-H5N1 prevalence rates in six epidemic waves ***(EW (1–6))*** in the backyard poultry ***(B2)*** in Menoufia, Egypt due to detection of sickly poultry by passive and/or active surveillance was examined **(**Table 5**)**. The effect of active and passive surveillance in the backyard poultry of three different types (domestic, mixed, and reservoir) were compared. In domestic chickens, the active surveillance had a high impact in decreasing the prevalence rates by 16, and 1% in both mixed and reservoir poultry (correlation coefficients = − 0.16, − 0.01) respectively. Whereas the passive surveillance had less impact in decreasing prevalence rates by 14% in mixed poultry and 3% in domestic chickens (correlation coefficients = − 0.14, and − 0.03 respectively). Therefore, in this study, active and passive surveillance were considered to be strong predictors of the likelihood in decrease HPAI-H5N1 prevalence rates in different poultry types not detected by passive or active surveillance in the backyard poultry ***(B2)***.

**Table 5:** Sensitivity ranking for the likelihood of HPAI-H5N1 prevalence rate in different poultry types not detected by surveillance in six epidemic waves in the backyard poultry (B2)

Sensitivity Ranking	Variables	Correlation coefficient values
1	*The probability of HPAI-H5N1 Prevalence rates in reservoir poultry in backyard*	0.80
2	*The probability of HPAI-H5N1 Prevalence rates in mixed poultry in backyard*	0.63
3	*The probability of HPAI-H5N1 Prevalence rates in domestic chicken in backyard*	0.60
4	*The probability of HPAI-H5N1 Prevalence rates in Backyard (B2) in each EW (Domestic chicken)*	0.46
	*The probability of HPAI-H5N1 Prevalence rates in Backyard (B2) in each EW (Mixed poultry)*	0.40
	*The probability of HPAI-H5N1 Prevalence rates in Backyard (B2) in each EW (Reservoir poultry)*	0.37
5	*The probability of HPAI-H5N1 prevalence rates in poultry in each epidemic wave (Domestic chicken)*	0.28
	*The probability of HPAI-H5N1 prevalence rates in poultry in each epidemic wave (Mixed poultry)*	0.25
	*The probability of HPAI-H5N1 prevalence rates in poultry in each epidemic wave (Reservoir poultry)*	0.22
6	*The probability of H5N1 Prevalence rates in domestic chicken due to failure in detection by active surveillance in backyard*	−0.16
7	*The probability of H5N1 Prevalence rates in mixed poultry due to failure in detection by passive surveillance in backyard*	−0.14
8	*The probability of H5N1 Prevalence rates in domestic chicken due to failure in detection by passive surveillance in backyard*	−0.03
9	*The probability of H5N1 Prevalence rates in mixed poultry due to failure in detection by active surveillance in backyard*	−0.01
10	*The probability of H5N1 Prevalence rates in reservoir poultry due to failure in detection by active surveillance in backyard*	−0.01
11	*The probability of H5N1 Prevalence rates in reservoir poultry due to failure in detection by passive surveillance in backyard*	0.00

**Fig. 5:**
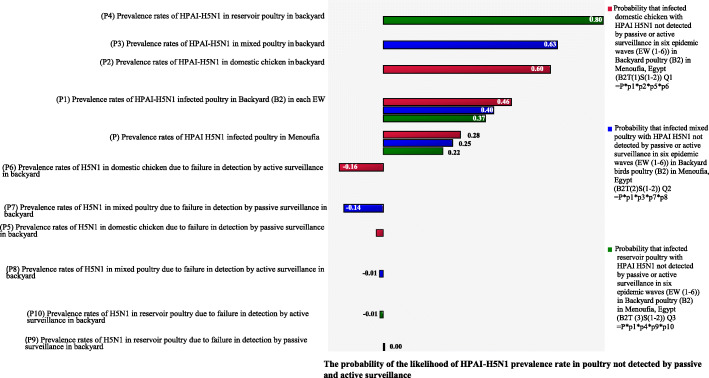
Tornado graph showing the likelihood of HPAI-H5N1 prevalence rate in different poultry types not detected by surveillance in six epidemic waves in the backyard poultry (B2)

Figure 5 shows the results of sensitivity analysis presented as the correlation coefficient of the overlaid tornado graphs, which provide a pictorial representation of HPAI-H5N1 prevalence rates in different poultry types not detected by passive and/or active surveillance in six epidemic waves ***(EW (1–6))*** in backyard poultry ***(B2)*** in Menoufia, Egypt. The sensitivity analysis illustrates the degree to which the uncertainties of output variables are affected by the uncertainty of the individual variables within the input variable. The higher the degree of correlation between the input and output variables (calculated using rank-order correlation), the more the input variable is affecting the output variable. The final output is affected by the following parameters by the same mentioned sequence: HPAI-H5N1 prevalence rates in reservoir poultry ***(P4)***; prevalence rates in mixed poultry ***(P3)***; prevalence rates in domestic chickens ***(P2)***; prevalence rates of all infected poultry in the backyard ***(P1)***; and prevalence rates of infection in poultry in each ***EW (P)***. The effect of active and passive surveillance as mitigation strategies in backyard poultry’s different types (domestic, mixed and reservoir) was compared. In domestic chickens, the active surveillance had a high impact in decreasing the HPAI-H5N1 prevalence rates by 16, and 1% in both mixed and reservoir poultry (correlation coefficients = − 0.16, − 0.01) respectively. Whereas the passive surveillance had less impact in decreasing prevalence rates by 14% in mixed poultry and 3% in domestic chickens (correlation coefficients = − 0.14, and − 0.03 respectively) **(**Table 5 and Fig. 5**)**.

## Discussion

The results of this study demonstrate that the likelihood of poultry still having a high prevalence rate of HPAI-H5N1 after failure of mitigation strategies such as vaccination, passive and active surveillance in six epidemic waves ***(EW (1–6))*** in the poultry farms ***(B1)*** in Menoufia, Egypt is still causing for alarm. In 90% of the time during Monte Carlo simulations, the expected probability of HPAI H5N1 prevalence in birds in farms is 48% due to failure of mitigation measures. Obtained results are broadly consistent with previous studies E Abdelwhab and H Hafez [11].

Probability distributions of HPAI-H5N1 prevalence rates in the poultry farms imply that coverage by vaccination and surveillance is unexpectedly low. HPAI-H5N1 prevalence rates in poultry in each epidemic wave (***P***) is about 37%, the probability that these infected poultry were vaccinated in farms (***P1***) is 3.6%. Although Egypt is considered the second country after China, accounting for over 99% of vaccination usage [19], surveys of vaccination coverage rates showed vaccination rates of less than 20% in sector 4 [19, 46], suggesting that the average coverage rate would be much lower than the calculated coverage [19]. Inadequate coverage of vaccinations makes the control of the virus spread difficult [19] because reducing HPAI-H5N1 viral load and transmission requires maintenance of high levels of flock immunity [18]. The low vaccination coverage could be attributed to inherent logistical problems in applying any type of vaccine since there are more poultry within sectors 3 and 4 where most of the production in Egypt takes place, which have more premises and independent management systems [19].

The probability that active and passive surveillance can detect those vaccinated poultry on the farms if they get infected is 0.27 and 0.09% respectively. This is because the Egyptian General Organization of Veterinary Services monitors less than 6.5% of vaccinated poultry in the commercial sectors [1, 12]. Field outbreaks have occurred in vaccinated countries, primarily because of inadequate coverage in the target species. Besides, in Egypt vaccine failures have occurred following antigenic drift in field viruses [22]. The immune pressure exerted by the vaccines or natural infection accelerates virus evolution in poultry, reducing the efficacy of vaccination over time [23, 32–37]. Moreover, improper administration, mishandling [38] and inappropriate storage of the vaccine [12] or suppression of the immune system (i.e.: due to chicken anemia virus infection or ingestion of mycotoxins), further reduces vaccination efficacy [39]. These could be considered as missed vaccinated poultry. In this study, the model predicts that HPAI-H5N1 prevalence rates in the poultry farms will likely increase to 70% in the case of missed vaccinated poultry (population still at risk after application of mass vaccination).

On the other hand, in the backyard poultry, the likelihood of still having a high HPAI-H5N1 prevalence rate in three different poultry types (domestic, mixed, and reservoir) due to failure of passive and active surveillance, varies between these three types. In mixed poultry, the likelihood of HPAI-H5N1 prevalence rates not detected by passive or active surveillance was higher with a mean of 16.8 × 10^− 3^. This was followed by domestic chickens with a mean of 1.48 × 10^− 3^ and reservoir poultry with a mean of 4.94 × 10^− 3^. In case of failure in detection of HPAI-H5N1 by passive and active surveillance in both the farm and backyard poultry, the prevalence rates will likely increase to 99%. Despite the high prevalence rates of HPAI-H5N1, with more infected poultry, surveillance were able to detect more infected flocks. However, we found that the effect of surveillance gets lower with high prevalence. This is an example of detection bias due to a decline in reporting due to reduced compliance from poultry owners after culling policies. This finding was expected because a previous studies reported that since the use of mass vaccination in Egypt, a decrease in disease incidence has been observed [1]. This may be because of the result of lower outbreak detection and inadequate reporting rather than immunity conferred by vaccination M Peyre, H Samaha, YJ Makonnen, A Saad, A Abd-Elnabi, S Galal, T Ettel, G Dauphin, J Lubroth and F Roger [1].

Overall, this study demonstrated that an increase in the completeness of poultry vaccination, active and passive surveillance of HPAI-H5N1 in the farms and backyard poultry in Menoufia, Egypt would have a decrease in the infection rates and ultimately decrease in HPAI-H5N1 prevalence rates. Consequently, sensitivity analysis results are to show the aggregate effect of each input variable has on a corresponding output variable -the likelihood of HPAI-H5N1 prevalence rates not detected by passive or active surveillance- together with their sensitivity ranking. The tornado graphs, which provide a pictorial representation of a sensitivity analysis of simulation results, which demonstrate the significance of each input variable has on an output variable.

In the poultry farms, it was indicated that an increase in efficacy of vaccination would lead to decrease of the likelihood of infection with HPAI-H5N1 however, with failure of mitigation strategies including failure to immunize poultry, Increase probability of infection. Will be observed. Increasing vaccination by one standard deviation (SD) will decrease the prevalence rates by only 14%, which is a limited effect as a primary required control measure in the mitigation strategy revealing how much vaccination faces a lot of challenges and limitations. One of the facts that cannot be ignored is that vaccination cannot prevent infection, or its transmission [47–50]. Besides circulation of the virus within vaccinated poultry [49, 51] along with inadequate vaccination coverage [18] and acceleration of viral evolution due to inadequate vaccination [17], in addition to improper antigenic matching between vaccines’ seed strain and circulating viruses [9].

Vaccination alone is not enough to reduce prevalence rates and infection spread. This should be integrated with other measures principally effective surveillance systems and outbreak management [1, 23]. Active to a more extent than passive surveillance is the second and third intervention strategies on the poultry farms. In this study, the effects of both active and passive surveillance as mitigation strategies in the backyard poultry’s different types (domestic, mixed and reservoir) are compared. Active surveillance had a higher impact in decreasing prevalence rates in domestic and mixed poultry by 16 and 14%, respectively. Whereas the active surveillance had less impact in decreasing prevalence rates by 3% in domestic chicken and 1% in both mixed and reservoir poultry.

It is not surprising that passive surveillance has less impact in detecting the likelihood of HPAI-H5N compared to active surveillance since farmers don’t report outbreaks neither in backyard nor commercial farms [52]. Generally, adherence to control measures is low because of social norms, especially in rural areas, and declining public awareness in the community [5]. The results reflect the true situation because of the prevailing under-reporting in the commercial sector to protect their business interests and prevent the mass culling of their stocks [53]. As well as, surveillance activities’ biases towards the household sector due to lack of incentive to report outbreaks as there is very limited or no compensation scheme for poultry owners [1, 10]. Besides, owners of the poultry farms are becoming reluctant to vaccinate their poultry or notify authorities if there is any infection after sudden deaths in poultry following vaccination campaigns “post-vaccination sudden death” [1]. In addition to that, local veterinary services consequently are reluctant to declare new outbreaks due to the fear of being unfairly blamed for failing to effectively perform their duties [1].

Nevertheless, some studies support the hypothesis that mass avian influenza vaccination of domestic chicken may negatively affect passive surveillance [1, 43], as it increases the viral load in the environment which leads to the possible presence of silent infection with masking clinical expression of the disease [54]. Not only vaccination could change the clinical features of the disease, but also concurrent infection by low-pathogenic avian influenza viruses can protect from disease signs and death if becoming infected with the HPAI-H5N1 [55], but still could shed HPAI-H5N1virus in their feces and could be capable of infecting other poultry, and potentially humans [56, 57].

Unlike Passive surveillance, which has many limitations, active surveillance is better than passive surveillance in detecting the virus as it is performed by highly experienced persons and more advanced techniques to detect even silent infected poultry. Increases in the surveillance activities (active) in form of utilization of rapid antigen detection test nationwide at the public veterinary clinics and the implementation of participating disease search (Community-Based Animal Health And Outreach Program-CAHO) enhanced the surveillance programs (active) leading to observed increases in reported cases in the household poultry during 2010/2011 [40]. It allows monitoring the evolution and prevalence of endemic viruses, which provide an early alert for the incursion of emerging viruses [5].

The results of this study are comparable with A Arafa, I El-Masry, S Khoulosy, MK Hassan, M Soliman, OG Fasanmi, FO Fasina, G Dauphin, J Lubroth and YM Jobre [40] study which has shown that more than 52% incidence rate was reported in the mixing of different bird species in the household sector in comparison to any other sector. The virus was more prevalent in mixed waterfowls and chickens than turkeys and pigeons [11]. Surveillance also indicated the persistence of H5N1 in scavenging household ducks where the contact with feral birds is higher than other poultry [58]. Ducks were speculated to be a major source for infection as the emergence of the new 2.2.1.2 cluster in the recent 2014/2015 upsurge in poultry and humans in Egypt where the predecessor virus of this clade was probably of a duck-origin virus [59]. This clade was responsible for a majority of documented human infections in recent years [60, 61]. Besides, viruses isolated from ducks were more genetically diverse than those isolated from chickens [62], suggesting a role for ducks in perpetuating the endemicity of A/H5N1 in Egypt. In this study, the model in the backyard poultry, demonstrated that decreasing HPAI-H5N1 infection in reservoir and mixed poultry might directly decrease the overall prevalence rates. Therefore, targeted surveillance to elucidate the spread of the HPAI-H5N1 virus in ducks and household with mixed different poultry species should be considered.

## Conclusion

It can be concluded from the quantitative risk assessment (QRA) model that the applied strategies were not sufficient to control the spread of the HPAI-H5N1 virus, but data quality, state of the scientific knowledge, and values of the parameters used to fit the model are continuously evolving. This calls for the need to periodically revise the QRA to update the estimates and improve the quality of the conclusions. The choice of an effective strategy will vary depending on an evaluation of the chosen mitigation strategy for combating outbreaks in the whole of Egypt. This QRA model dynamics are to be applied with other data if it becomes available. The lack of inclusion of detailed biosecurity control measures that weren’t recorded, is a limitation of this study. Although, this is a model for avian influenza HPAI-H5N1 strain, the nature of the next epidemic virus strain is uncertain. The model could also apply to most infectious poultry diseases especially those of viral nature. The effectiveness of any strategy depends mainly on its rapid implementation and a strong political commitment, along with a strict poultry owners’ level of compliance. Using the HPAI-H5N1 virus QRA model can also help public health officials to take into consideration the evaluation of the mitigations and control strategies in their response to this disease of great public health importance. Although this QRA is developed for the Menoufia, governorate, Egypt, it is generic in nature and it can be applied in areas with similar prevailing problem.

## Supplementary Information

**Additional file 1.**

## Data Availability

All data generated or analyzed during this study are included in this published article [and its supplementary information files.

## References

[1] Peyre M, Samaha H, Makonnen YJ, Saad A, Abd-Elnabi A, Galal S, Ettel T, Dauphin G, Lubroth J, Roger F (2009). Avian influenza vaccination in Egypt: limitations of the current strategy. Journal of Molecular and Genetic Medicine: An International Journal of Biomedical Research.

[2] Salaheldin AH, Abd El-Hamid HS, Elbestawy AR, Veits J, Hafez HM, Mettenleiter TC, Abdelwhab EM (2018). Multiple introductions of influenza a (H5N8) virus into poultry, Egypt, 2017. Emerg Infect Dis.

[3] Hagag NM, Erfan AM, El-Husseiny M, Shalaby AG, Saif MA, Tawakol MM, Nour AA, Selim AA, Arafa A-S, Hassan MK (2019). Isolation of a novel reassortant highly pathogenic avian influenza (H5N2) virus in Egypt. Viruses.

[4] El-Zoghby EF, Arafa A-S, Hassan MK, Aly MM, Selim A, Kilany WH, Selim U, Nasef S, Aggor MG, Abdelwhab E (2012). Isolation of H9N2 avian influenza virus from bobwhite quail (Colinus virginianus) in Egypt. Arch Virol.

[5] El-Shesheny R, Kandeil A, Mostafa A, Ali MA, Webby RJ. H5 Influenza Viruses in Egypt. Cold Spring Harbor Perspectives in Medicine. 2020:a038745. 10.1101/cshperspect.a038745. 10.1101/cshperspect.a038745 PMC8168528 32122919

[6] Chen L-M, Blixt O, Stevens J, Lipatov AS, Davis CT, Collins BE, Cox NJ, Paulson JC, Donis RO (2012). In vitro evolution of H5N1 avian influenza virus toward human-type receptor specificity. Virology.

[7] Schmier S, Mostafa A, Haarmann T, Bannert N, Ziebuhr J, Veljkovic V, Dietrich U, Pleschka S (2015). In silico prediction and experimental confirmation of HA residues conferring enhanced human receptor specificity of H5N1 influenza a viruses. Sci Rep.

[8] WHO. Cumulative number of confirmed human cases for avian influenza A (H5N1) reported to WHO, 2003–2019. WHO, Geneva 2019:3.

[9] Kayali G, Kandeil A, El-Shesheny R, Kayed AS, Maatouq AM, Cai Z, McKenzie PP, Webby RJ, El Refaey S, Kandeel A (2016). Avian influenza a (H5N1) virus in Egypt. Emerg Infect Dis.

[10] Kayali G, Webby RJ, Ducatez MF, El Shesheny RA, Kandeil AM, Govorkova EA, Mostafa A, Ali MA (2011). The epidemiological and molecular aspects of influenza H5N1 viruses at the human-animal interface in Egypt. PLoS One.

[11] Abdelwhab E, Hafez H (2011). An overview of the epidemic of highly pathogenic H5N1 avian influenza virus in Egypt: epidemiology and control challenges. Epidemiology & Infection.

[12] Hafez M, Arafa A, Abdelwhab E, Selim A, Khoulosy S, Hassan M, Aly M (2010). Avian influenza H5N1 virus infections in vaccinated commercial and backyard poultry in Egypt. Poult Sci.

[13] Aly M, Arafa A, Hassan M (2008). Epidemiological findings of outbreaks of disease caused by highly pathogenic H5N1 avian influenza virus in poultry in Egypt during 2006. Avian Dis.

[14] Abdelwhab E, Selim A, Arafa A, Galal S, Kilany W, Hassan M, Aly M, Hafez M (2010). Circulation of avian influenza H5N1 in live bird markets in Egypt. Avian Dis.

[15] Arafa A, Suarez D, Hassan M, Aly M (2010). Phylogenetic analysis of hemagglutinin and neuraminidase genes of highly pathogenic avian influenza H5N1 Egyptian strains isolated from 2006 to 2008 indicates heterogeneity with multiple distinct sublineages. Avian Dis.

[16] Eladl AEH, El-Azm KIA, Ismail AEN, Ali A, Saif YM, Lee C-W (2011). Genetic characterization of highly pathogenic H5N1 avian influenza viruses isolated from poultry farms in Egypt. Virus Genes.

[17] Cattoli G, Fusaro A, Monne I, Coven F, Joannis T, El-Hamid HSA, Hussein AA, Cornelius C, Amarin NM, Mancin M (2011). Evidence for differing evolutionary dynamics of A/H5N1 viruses among countries applying or not applying avian influenza vaccination in poultry. Vaccine.

[18] El Masry I, Rijks J, Peyre M, Taylor N, Lubroth J, Jobre Y (2014). Modelling influenza A H5N1 vaccination strategy scenarios in the household poultry sector in Egypt. Trop Anim Health Prod.

[19] Swayne D, Pavade G, Hamilton K, Vallat B, Miyagishima K (2011). Assessment of national strategies for control of high-pathogenicity avian influenza and low-pathogenicity notifiable avian influenza in poultry, with emphasis on vaccines and vaccination. Revue Scientifique et Technique-OIE.

[20] EMPRES/GLEWS: H5N1 HPAI Global Overview **-** April 2009**.** Food and agriculture organisation, emergency prevention systems and global early warning system for major animal diseases HPAI bulletin**.** Issue No 10**.** 2009.

[21] Domenech J, Dauphin G, Rushton J, McGrane J, Lubroth J, Tripodi A, Gilbert J, Sims L (2009). Experiences with vaccination in countries endemically infected with highly pathogenic avian influenza: the food and agriculture organization perspective. Rev Sci Tech.

[22] Swayne DE (2012). Impact of vaccines and vaccination on global control of avian influenza. Avian Diseases.

[23] Abdelwhab E, Hassan M, Abdel-Moneim A, Naguib M, Mostafa A, Hussein I, Arafa A, Erfan A, Kilany W, Agour M (2016). Introduction and enzootic of A/H5N1 in Egypt: virus evolution, pathogenicity and vaccine efficacy ten years on. Infect Genet Evol.

[24] Habtemariam T (1989). Utility of epidemiologic simulation models in the planning of trypanosomiasis control programs. Ann Soc Belg Med Trop.

[25] Abdalla E, HabteMariam T, Nganwa D, Dibaba AB, Gerbi G, Vinaida R, Tameru B (2011). Epidemiology of influenza A 2009 H1N1 virus pandemic in the US. J Health Care Poor Underserved.

[26] Nganwa D, Habtemariam T, Tameru B, Gerbi G, Bogale A, Robnett V, Wilson W (2010). Applying the epidemiologic problem oriented approach (EPOA) methodology in developing a knowledge base for the modeling of HIV/AIDS. Ethn Dis.

[27] Habtemariam T, Tameru B, Ahmad A, Nganwa D, Ayanwale L, Beyene G, Robnett V. The role of risk assessment in managing risk. In*.*; 2005.

[28] Murray N. Import risk analysis: animals and animal products Import risk analysis: animals and animal products 2002.

[29] Elsobky Y, El Afandi G, Abdalla E, Byomi A, Reddy G (2020). Possible ramifications of climate variability on HPAI-H5N1 outbreak occurrence: case study from the Menoufia, Egypt. PLoS One.

[30] Palisade. Palisade Knowledge Base. Retrieved from: https://kb.palisade.com/index.php?pg=kb.page&id=1686. 2018.

[31] Toma B, Dufour B, Sanaa M, Benet J, Moutou F, Louza A, Ellis P. Applied veterinary epidemiology and the control of disease in populations: association pour l'Etude de l'Epidémiologie des Maladies Animales, 7 Avenue…; 1999.

[32] Cattoli G, Milani A, Temperton N, Zecchin B, Buratin A, Molesti E, Aly MM, Arafa A, Capua I (2011). Antigenic drift in H5N1 avian influenza virus in poultry is driven by mutations in major antigenic sites of the hemagglutinin molecule analogous to those for human influenza virus. J Virol.

[33] Abdel-Moneim AS, Afifi MA, El-Kady MF (2011). Genetic drift evolution under vaccination pressure among H5N1 Egyptian isolates. Virol J.

[34] Abdelwhab E, Arafa A-S, Stech J, Grund C, Stech O, Graeber-Gerberding M, Beer M, Hassan MK, Aly MM, Harder TC (2012). Diversifying evolution of highly pathogenic H5N1 avian influenza virus in Egypt from 2006 to 2011. Virus Genes.

[35] Grund C, Abdelwhab E-SM, Arafa A-S, Ziller M, Hassan MK, Aly MM, Hafez HM, Harder TC, Beer M (2011). Highly pathogenic avian influenza virus H5N1 from Egypt escapes vaccine-induced immunity but confers clinical protection against a heterologous clade 2.2. 1 Egyptian isolate. Vaccine.

[36] Lee C-W, Senne DA, Suarez DL (2004). Effect of vaccine use in the evolution of Mexican lineage H5N2 avian influenza virus. J Virol.

[37] Escorcia M, Vázquez L, Méndez ST, Rodríguez-Ropón A, Lucio E, Nava GM (2008). Avian influenza: genetic evolution under vaccination pressure. Virol J.

[38] El-Zoghby EF, Arafa A-S, Kilany WH, Aly MM, Abdelwhab EM, Hafez HM (2012). Isolation of avian influenza H5N1 virus from vaccinated commercial layer flock in Egypt. Virol J.

[39] Hegazy A, Abdallah F, Abd-El-Samie L, Nazim A (2011). The relation between some immunosuppressive agents and widespread nature of highly pathogenic avian influenza (HPAI) post vaccination. J Am Sci.

[40] Arafa A, El-Masry I, Khoulosy S, Hassan MK, Soliman M, Fasanmi OG, et al. Predominance and geo-mapping of avian influenza H5N1 in poultry sectors in Egypt. Geospat Health. 2016;11(3). 10.4081/gh.2016.492. 10.4081/gh.2016.492 27903065

[41] El-Zoghby EF, Aly MM, Nasef SA, Hassan MK, Arafa A-S, Selim AA, Kholousy SG, Kilany WH, Safwat M, Abdelwhab E (2013). Surveillance on a/H5N1 virus in domestic poultry and wild birds in Egypt. Virol J.

[42] ElMasry I, Elshiekh H, Abdlenabi A, Saad A, Arafa A, Fasina FO, Lubroth J, Jobre Y (2017). Avian influenza H5N1 surveillance and its dynamics in poultry in live bird markets, Egypt. Transbound Emerg Dis.

[43] Vergne T, Grosbois V, Jobre Y, Saad A, El Nabi AA, Galal S, Kalifa M, El Kader SA, Dauphin G, Roger F (2012). Avian influenza vaccination of poultry and passive case reporting, Egypt. Emerg Infect Dis.

[44] Palisade. Risk Analysis “Stochastic Risk Analysis - Monte Carlo Simulation”. Retrieved from: https://www.palisade.com/risk/risk_analysis.asp. 2020.

[45] Gerbi GB, Habtemariam T, Tameru B, Nganwa D, Robnett V (2012). A quantitative risk assessment of multiple factors influencing HIV/AIDS transmission through unprotected sex among HIV-seropositive men. AIDS Care.

[46] Egypt Go. Integrated National Plan for avian and human influenza. Animal health and livelihood sustainability strategy. Ministry of Agriculture and Land Reclamation, Cairo, Egypt, 1–27**.** 2010.

[47] Capua I, Marangon S (2004). Vaccination for avian influenza in Asia. Vaccine.

[48] Cardona CJ, Charlton BR, Woolcock PR (2006). Persistence of immunity in commercial egg-laying hens following vaccination with a killed H6N2 avian influenza vaccine. Avian Dis.

[49] Savill NJ, St Rose SG, Keeling MJ, Woolhouse ME (2006). Silent spread of H5N1 in vaccinated poultry. Nature.

[50] Van der Goot J, Van Boven M, de Jong MCM, Koch G (2007). Effect of vaccination on transmission of HPAI H5N1: the effect of a single vaccination dose on transmission of highly pathogenic avian influenza H5N1 in Peking ducks. Avian Dis.

[51] Hulse-Post D, Sturm-Ramirez K, Humberd J, Seiler P, Govorkova E, Krauss S, Scholtissek C, Puthavathana P, Buranathai C, Nguyen T (2005). Role of domestic ducks in the propagation and biological evolution of highly pathogenic H5N1 influenza viruses in Asia. Proc Natl Acad Sci.

[52] Rijks J, ElMasry I. Characteristics of poultry production in Egyptian villages and their effect on HPAI vaccination campaign results–results of a participatory epidemiology study. Food and Agriculture Organisation Report. 2009.

[53] FAO. Approaches to controlling, preventing and eliminating H5N1 Highly Pathogenic Avian Influenza in endemic countries. 2011. FAO., http://www.fao.org/docrep/014/i2150e/i2150e.pdf. 2011.

[54] Martin V, Pfeiffer DU, Zhou X, Xiao X, Prosser DJ, Guo F, Gilbert M (2011). Spatial distribution and risk factors of highly pathogenic avian influenza (HPAI) H5N1 in China. PLoS Pathog.

[55] Webster RG, Peiris M, Chen H, Guan Y (2006). H5N1 outbreaks and enzootic influenza. Biodiversity.

[56] Seo SH, Webster RG (2001). Cross-reactive, cell-mediated immunity and protection of chickens from lethal H5N1 influenza virus infection in Hong Kong poultry markets. J Virol.

[57] O'Neill E, Seo SH, Woodland DL, Shortridge KF, Webster RG. Infection with H9N2 influenza viruses confers immunity against lethal H5N1 infection. In: International Congress Series: 2001: Elsevier; 2001: 775–781.

[58] Sheta BM, Fuller TL, Larison B, Njabo KY, Ahmed AS, Harrigan R, Chasar A, Aziz SA, Khidr A-AA, Elbokl MM (2014). Putative human and avian risk factors for avian influenza virus infections in backyard poultry in Egypt. Vet Microbiol.

[59] Arafa A, Naguib M, Luttermann C, Selim A, Kilany W, Hagag N, Samy A, Abdelhalim A, Hassan M, Abdelwhab E (2015). Emergence of a novel cluster of influenza A (H5N1) virus clade 2.2. 1.2 with putative human health impact in Egypt, 2014/15. Eurosurveillance.

[60] WHO/OIE/FAO (2012). Continued evolution of highly pathogenic avian influenza A (H5N1): updated nomenclature. Influenza Other Respir Viruses.

[61] Arafa A, El-Masry I, Kholosy S, Hassan MK, Dauphin G, Lubroth J, Makonnen YJ (2016). Phylodynamics of avian influenza clade 2.2. 1 H5N1 viruses in Egypt. Virol J.

[62] Watanabe Y, Ibrahim MS, Ellakany HF, El-Hamid HSA, Ikuta K (2011). Genetic diversification of H5N1 highly pathogenic avian influenza a virus during replication in wild ducks. J Gen Virol.

